# Effect of Adding Al on the Phase Structure and Gettering Performance of TiZrV Non-Evaporable Getter Materials

**DOI:** 10.3390/ma17091969

**Published:** 2024-04-24

**Authors:** Lulu Wang, Yang Li, Deyu Guo, Qingxi Jin, Zhenbin Zhang, Zhimin Yang

**Affiliations:** 1 State Key Laboratory for Advanced Materials for Smart Sensing, GRINM Group Co., Ltd., Beijing 100088, China; 15953284537@163.com (L.W.); liyang@grinm.com (Y.L.); jinqingxi@foxmail.com (Q.J.); z18716026882@163.com (Z.Z.); 2 GRIMAT Engineering Institute Co., Ltd., Beijing 101407, China; 3 General Research Institute for Nonferrous Metals, Beijing 100088, China

**Keywords:** NEG, TiZrV, in situ XPS, phase

## Abstract

Titanium zirconium vanadium (TiZrV) is a widely used non-evaporable getter (NEG) material with the characteristics of a low activation temperature and a large gas absorption capacity. At present, the research on TiZrV getters mainly focuses on the thin-film state, with little research on the bulk state. In this paper, a TiZrV getter was optimized by adding Al, and the phase structure, activation properties, and gettering performance were studied. With the addition of Al, the α-Zr phase and Ti_2_Zr phase changed into the Ti-Zr phase and Al-Zr, Al-Ti phase. The newly generated phase promoted the diffusion of hydrogen and oxygen atoms. The activation temperature decreased significantly, as shown in the in situ XPS results. The H_2_ and CO gettering performance of TiZrVAl samples was promoted to 2073 cm^3^·s^−1^ and 1912.8 cm^3^·s^−1^, increased by 40.7% and 40.3%. This paper provides valuable ideas for optimizing the properties of bulk TiZrV getters.

## 1. Introduction

NEG materials are widely used in the field of vacuum devices and vacuum apparatus. They are a kind of material that can absorb active gases such as hydrogen, oxygen, carbon oxides, and nitrogen under vacuum conditions [1]. They are mainly used to remove residual gas in vacuum systems, improve the vacuum degree of a system, maintain a good vacuum state, prolong the service life of vacuum devices, and improve the reliability of vacuum systems [2]. With the development of vacuum technology, NEG materials with superior gettering performance are required.

TiZrV is an advanced NEG material, commonly used in vacuum devices with a thin-film state. Benvenuti, C. et al. [3] prepared a thin TiZrV film that could be activated at 180 °C × 24 h by adjusting the composition. Šutara, F. et al. [4] monitored the valence changes of various alloy elements during the activation process with in situ XPS, and found that V is reduced first, followed by Ti, and Zr was the most difficult to reduce. At present, TiZrV getter films can be improved by adding alloy elements, and common added elements include Al, Cu, Hf, Cr, rare earth elements, etc. [5,6,7,8]. Thin-film getters and bulk getters have different preparation technologies, characteristics, and application scenarios [9,10]. Bulk getters are manufactured by powder metallurgy technology, and thin-film getters are obtained by vapor deposition [11,12]. The absorbed quantity of thin-film getters is limited; they cannot cope with high levels of impurity gases, and the effects of bulk getters in such a scenario is better. At present, common bulk getter materials include ZrVFe, TiZrV, TiMo, ZrAl, Zr graphite, etc. [13,14,15,16,17]; the activation temperature of TiZrV is the lowest among them. However, there is relatively little research on TiZrV bulk materials.

We have chosen a TiZrV material for further improvement in order to obtain a more highly performing bulk getter. We studied the effect of adding Al on the phase composition and gettering performance of a TiZrV bulk getter. It was found that the phase structure of the getter materials changed after doping Al, the activation temperature decreased, and the gettering performance was also significantly improved.

## 2. Materials and Methods

### 2.1. Preparation of the Bulk Getter

The high-purity material included Zr, Ti, V, and Al, which were purchased from Trillion Metals Co., Ltd., Beijing, China. The proportions of TiZrV and TiZrVAl samples 1–3# are shown in Table 1. The alloy ingots were prepared with suspension melting. Alloy powders were obtained by crushing the alloy ingot and sieving to −250 mesh. The samples to be characterized for gettering performance were pressed into a circular plate (pressure: 5 Mpa); 0.6 g of powder was used for each plate, whose dimension was Ø10 mm × 1 mm.

### 2.2. Characterization

X-ray photoelectron spectroscopy (XPS) on a ThermoFisher, Waltham, MA, USA, Thermo Scientific Nexsa Surface Analysis System was employed to characterize the surface state during the activation process. The X-ray source type is a monochrome, microfocused, low-power AlK-Alpha X-ray source. The X-ray optical spot size was 400 μm, and we analyzed at a pressure of 10^−7^ Pa. The samples were degassed at 100 °C for 3 h; the temperature was increased from 100 °C to 500 °C for 30 min at intervals of 100 °C. The element states of the sample were detected during the holding process.

The phase structure was characterized by the Rigaku, Tokyo, Japan, SmartLab SE (Cu target), with a voltage of 36 kV, a scanning speed of 2°/min, a test angle range of 10°~90°, and an operating current of 20 mA.

The microstructure was photographed by a JEOL, Tokyo, Japan, JSM-IT700HR field emission scanning electron microscope (FESEM); photos were taken using the JEOL, Tokyo, Japan, BSE-COMPO mode.

The sample sections necessary for TEM were prepared by ZEISS, Oberkochen, Germany, Crossbeam 540 focused ion beam SEM (FIB-SEM). Microstructural characterization of the sections was performed on a type FEI, Hillsboro, OR, USA, Tecnai F20 projection electron microscope (TEM/HRTEM), using a copper mesh.

The ICP-Ms was characterized by a ThermoFisher, Waltham, MA, USA, ICAP6300.

The gettering performance of the samples was tested according to GB/T 8763-2020 [18]. The whole system (except the sample chamber) needed to be heated at 180 °C for 3 h in order to remove the residual gas from the whole system. The activation conditions of the sample were 350 °C × 10 min for the H_2_ test and 500 °C × 10 min for the CO and N_2_ test. The test temperature of gettering H_2_ was 25 °C, and that of CO and N_2_ was 200 °C. The numerical value of the inhalation rate was calculated, and our instrument accuracy could only display up to the current order of magnitude of 0.1, so similar mantissa may have occurred during the calculation. The working characteristic of getter materials is their hydrogen absorption performance, which should be as high as possible, while their performance in absorbing CO and N_2_ will significantly decrease. Therefore, activation at 350 °C can achieve a significant difference in hydrogen performance, but the difference in CO and N_2_ absorption is relatively small. In order to highlight the effect of Al doping, the activation temperature and reaction temperature were increased. Therefore, we chose 500 °C as the activation temperature to test carbon monoxide and nitrogen. Due to the limitations of our testing equipment, the Pg was kept within an appropriate range. Excessive or insufficient suction rates can affect the testing accuracy. Therefore, we chose an operating temperature of 200 °C to test the gettering performance of CO and N_2_.

## 3. Results

**Figure 1:**
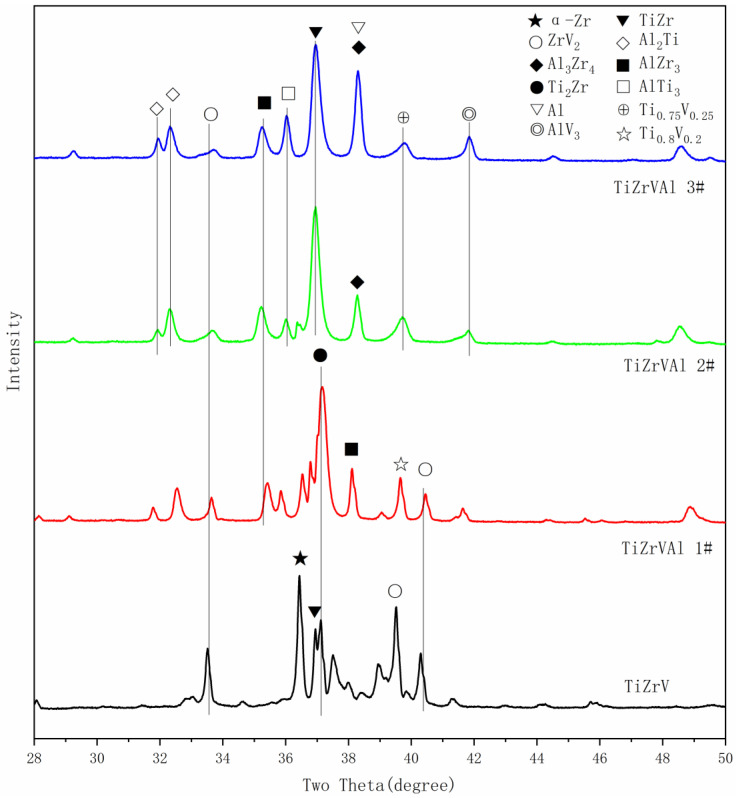
XRD results of the TiZrVAl 1#, 2#, and 3# and TiZrV samples.

**Figure 2:**
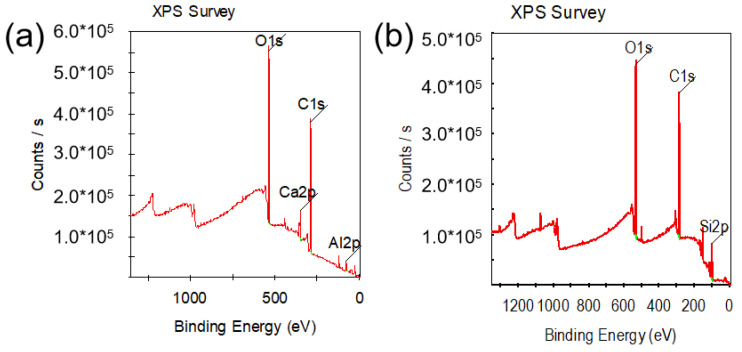
XPS results of silicon wafer with coating: (**a**) TiZrVAl 3#, (**b**) TiZrVAl 2#.

The phase structure of the tested samples is shown in Figure 1. The phase composition of the TiZrV samples mainly includes α-Zr, ZrV_2_, a TiZr phase, and a Ti_2_Zr phase [19]. With the addition of Al, the α-Zr phase disappeared, and the content of the ZrV_2_ phase decreased. In TiZrVAl sample 1#, the Ti_2_Zr phase and AlZr_3_ phase showed higher content, with a small amount of the Ti_0.8_V_0.2_ phase [20,21]. The phase composition of TiZrVAl 2# and 3# was relatively similar, displaying different contents. The phases mainly included a TiZr phase, an Al3Zr4 phase, and a small amount of the AlTi_3_ phase, Ti_0.75_V_0.25_ phase, and AlV_3_ phase [22,23]. The peak located at the position of 38.4° in TiZrVAl sample 3#’s pattern, related to the Al_3_Zr_4_ and Al phases, was significantly enhanced. To verify the presence of elemental Al, the surface temperature of TiZrVAl samples 2# and 3# were sustained at 500 °C for 1 h in a high vacuum environment by using ceramic heating plates. And a silicon wafer was placed above the sample to collect the Al vapor. According to the XPS results shown in Figure 2 and Table 2, the component of the gray impurity on the wafer for TiZrVAl 3# was Al. This is in harmony with the XRD results of TiZrVAl sample 3#. This indicates that the 11% Al addition could not fully react with the other elements to generate alloy phases. The small amount of Ca element in the XPS analysis may come from the impurities in the raw materials or the contamination generated during sample transferring and testing. The ICP-MS analysis was conducted on the composition of the raw materials, and the results in Table 3 show that there was a small amount of Ca element in the Zr raw materials.

**Figure 3:**
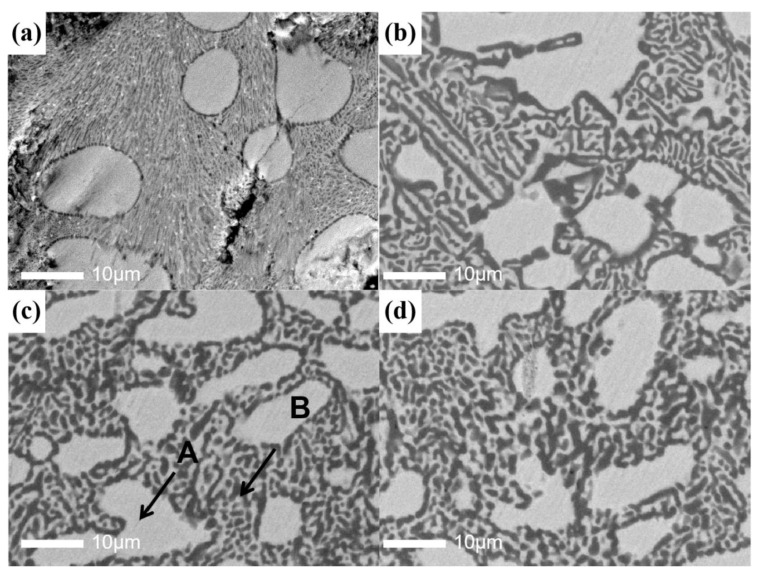
SEM images of the TiZrV and TiZrVAl 1–3# samples: (**a**) TiZrV, (**b**–**d**) TiZrVAl 1–3#.

Figure 3 shows the SEM images of all samples. Figure 3a shows the microstructure of TiZrV, including a large number of fine grains and a few large grains. With the addition of Al, the morphology of the large grains did not show a significant change; the small branch crystal tended to grow, a large number of the fine grains present in TiZrV disappeared, and many dendrites appeared. The further addition of Al had no more obvious effects on the crystal structure. Table 4 shows the components of dot A and dot B in TiZrVAl sample 2#. The large grains marked with dot A have higher Zr and Ti contents, which should be related to the AlZr_2_ phase and TiZr phase. The dot B section contains higher amounts of Al and V, corresponding to the Al-Ti, Al-Zr, Al-V, and Ti-V phases.

**Figure 4:**
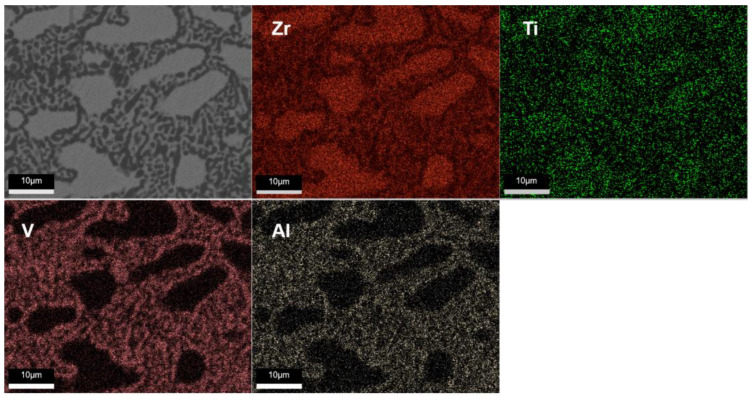
EDX images of TiZrVAl 2#.

Figure 4 shows the EDX pictures of TiZrVAl sample 2#. The Zr and Ti were distributed throughout the region, consistent with the intersoluble properties of Zr and Ti. The distribution of Al and V was relatively similar, opposite to the regions enriched in Ti and Zr. It should be an alloy phase consisting of four elements in the enriched regions of V and Al, matched with small branch crystal marked with dot B in Figure 3c. The other regions were dominated by a TiZr phase doped with Al and V, similar to the large crystal marked with A in Figure 3c.

**Figure 5:**
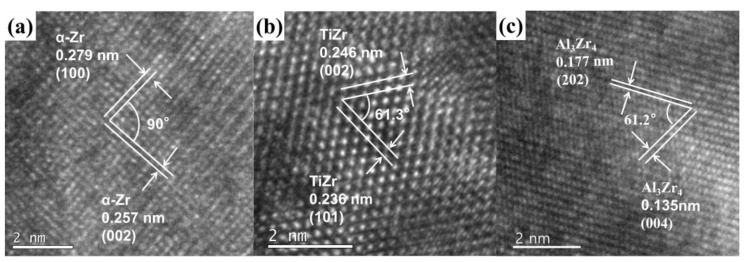
HRTEM images of samples: (**a**) white Zr-rich area of TiZrV, (**b**) white Zr-rich area of TiZrVAl 2#, and (**c**) black Al-rich area of TiZrVAl 2#.

Figure 5 shows the HRTEM images of the TiZrV sample and TiZrVAl sample 2#. The images captured the white Zr-rich area of TiZrV and TiZrVAl 2# shown in Figure 3a,b. Figure 3c shows the image of the black Al-rich area of TiZrVAl 2#. The area in the TiZrV sample is mainly composed of a TiZr phase, α-Zr, and a Ti_2_Zr phase. The phase is mainly composed of TiZr and an alloy phase of Al in TiZrVAl sample 2#.

In Figure 5a, the measured crystal plane spacing is 0.278 nm and 0.257 nm, respectively, which is close to their theoretical crystal plane spacing of 0.27988 nm and 0.25742 nm. And the two crystal planes with a 90° range were matched with the (100) and (002) crystal planes of the α-Zr phase.

In Figure 5b, the angle between them is about 61.3°, corresponding to the (002) and (101) crystal planes, with a crystal plane spacing of 0.246 nm and 0.236 nm, respectively. The interplanar spacing of the TiZr (002) crystal plane is 0.24505 nm, and the interplanar spacing of the (101) crystal plane is 0.23604 nm.

In Figure 5c, the measured crystal plane spacing is 0.177 nm and 0.135 nm, respectively, which is close to their theoretical crystal plane spacing of 0.17723 nm and 0.13475 nm. And the two crystal planes with a 90° range were matched with the (202) and (004) crystal planes of the Al_3_Zr_4_ phase.

**Figure 6:**
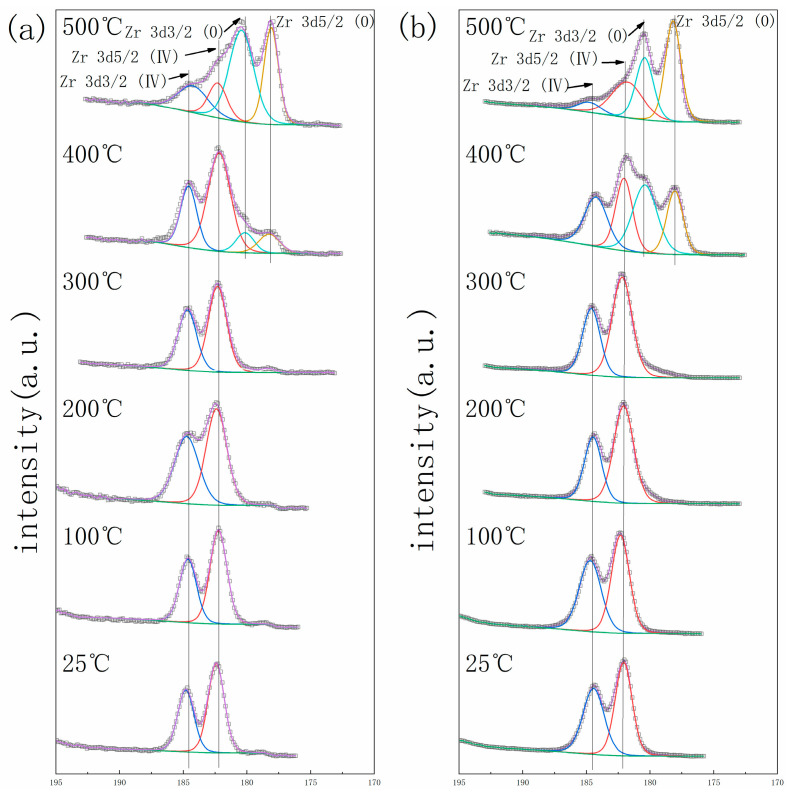
In situ XPS result of Zr: (**a**) TiZrV, (**b**) TiZrVAl 2#.

**Figure 7:**
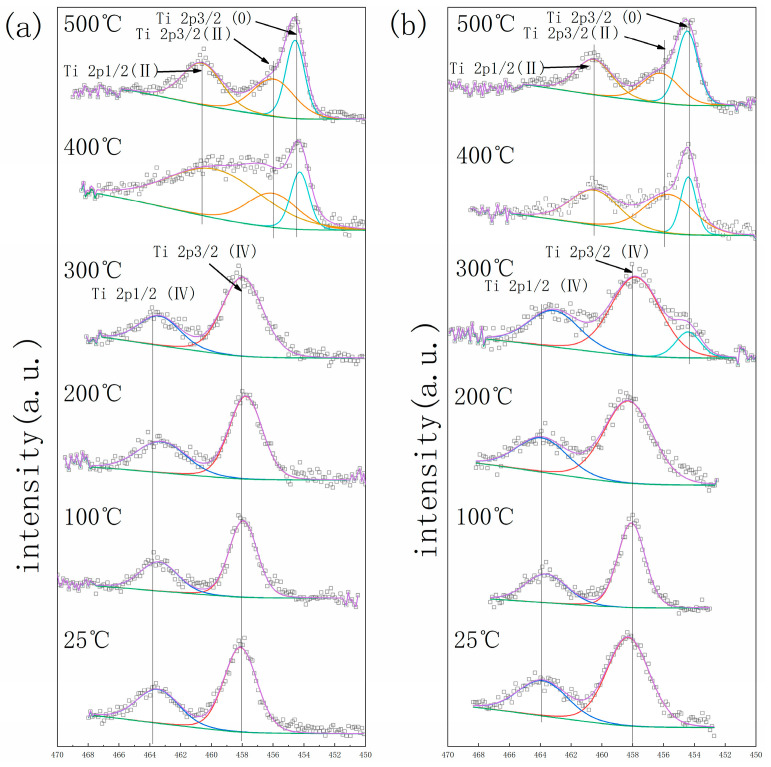
In situ XPS result of Ti: (**a**) TiZrV, (**b**) TiZrVAl 2#.

**Figure 8:**
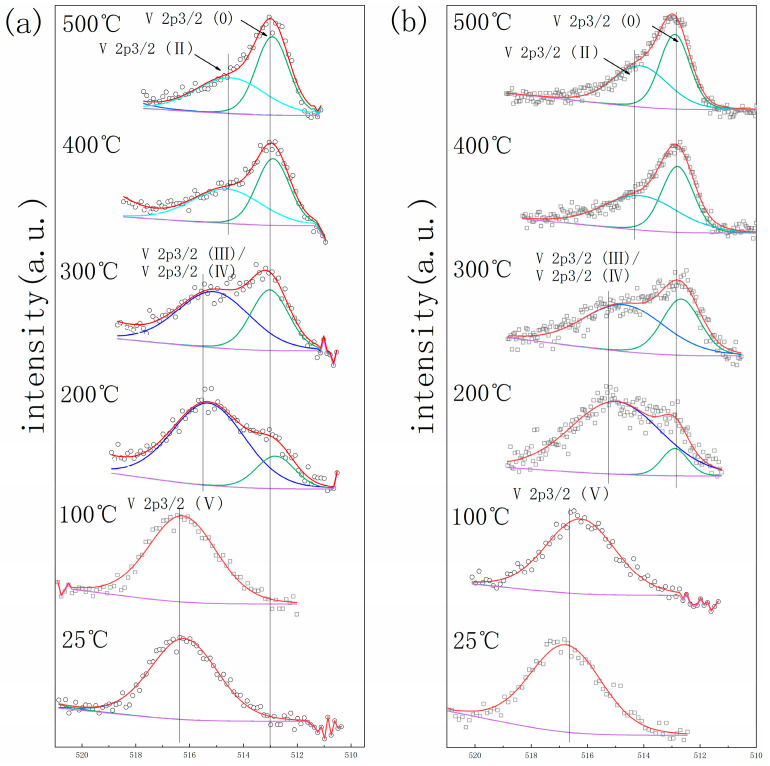
In situ XPS result of V: (**a**) TiZrV, (**b**) TiZrVAl 2#.

**Figure 9:**
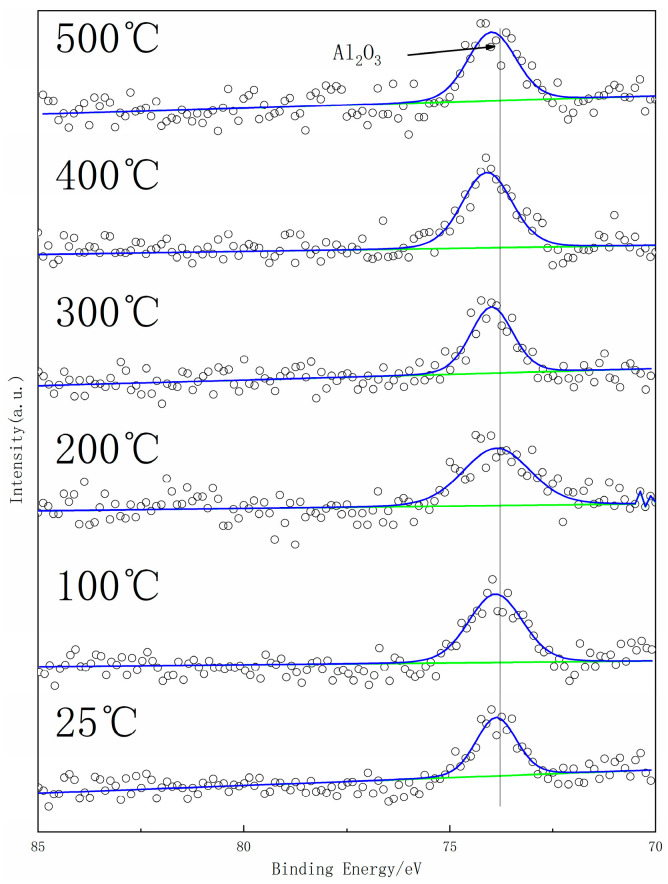
In situ XPS result of Al of TiZrVAl 2#.

The getter materials’ surface will form an oxide layer when exposed to the air, which leads to a deterioration in gettering performance. The activation process with an elevated temperature is needed to promote the passivation layer atoms’ diffusion into the bulk getter materials to acquire a fresh surface before it can work normally. In situ XPS was employed to monitor the oxidation state of metal elements on the surface of the getter material during the activation process [24]. The calibration of the XPS result was based on the C1s as 284.8 eV. Figure 6, Figure 7 and Figure 8 show the element spectra of TiZrV and TiZrVAl 2# at different activation temperatures. In Figure 6, the spectra of Zr 3*d*5/2 (0), Zr 3*d*3/2 (0), Zr 3*d*5/2 (Ⅳ), and Zr 3*d*3/2 (Ⅳ) peak at 178.7 eV, 181.1 eV, 182.4 eV, and 184.6 eV. The metallic state of Zr appeared clearly at 400 °C, which was shown with a higher content in TiZrVAl 2#. At 500 °C, significant metallic Zr peaks were observed at the TiZrV and TiZrVAl 2# samples simultaneously. In Figure 7, the spectra of Ti 2*p*3/2 (0), Ti 2*p*3/2 (Ⅱ), Ti 2*p*3/2 (Ⅳ), Ti 2*p*1/2 (Ⅱ), and Ti 2*p*1/2 (Ⅳ) peak at 454.5 eV, 456 eV, 458 eV, 460.3 eV, and 464 eV. The metal state of Ti in TiZrVAl 2# appeared at 300 °C, which was 100 °C lower than that of TiZrV. At 400 °C, the higher content of metallic Ti was shown in TiZrVAl 2#, in contrast to TiZrV. In Figure 8, the spectra of V 2*p*3/2 (0), V 2*p*3/2 (Ⅱ), V 2*p*3/2 (Ⅲ), V 2*p*3/2 (Ⅳ), and V 2*p*3/2 (Ⅴ) peak at 512.9 eV, 514.2 eV, 515.8 eV, 515.7 eV, and 516.6 eV [25]. The metal state of V in TiZrV and TiZrVAl 2# appeared at 200 °C. The metal-state proportions of V at 300 °C and 500 °C were close to each other. In Figure 9, the spectrum of the Al_2_O_3_ peak was at the position of 74.3 eV. The results indicate that Al_2_O_3_ was not affected by temperature. The results indicate that the element reduction order in the samples followed V > Ti > Zr, and the addition of Al contributed to a decrease in the starting reduction temperature of Ti and Zr. Since impurity molecules with chemisorption on the surface of a getter can hardly leave the alloy, the results of our in situ XPS analysis can represent the diffusion difficulty of O, C, and N and other elements to some extent, and have some reference value for the sorption speed prediction of the gas composition of these elements.

**Figure 10:**
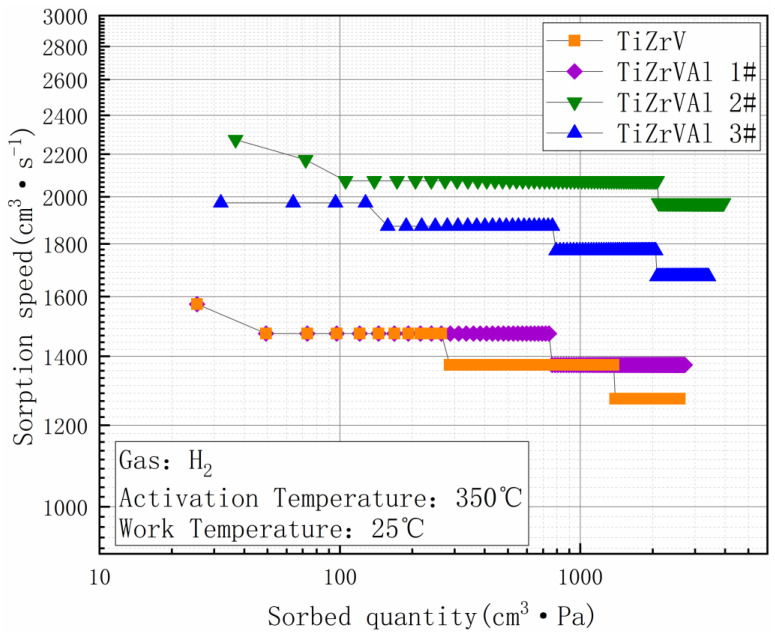
Performance of absorbing H_2_ of TiZrV and TiZrVAl 1–3#.

Figure 10 shows the H_2_ gettering performance of samples. The samples were activated at 350 °C, and their hydrogen absorption properties were tested at 25 °C. The sorption speed of TiZrVAl 1# was similar to that of TiZrV in the initial stage of the test, displaying a low decay rate. The initial sorption speed of the TiZrV sample at 10 min was 1473 cm^3^·s^−1^, and the final sorption speed at the end of the test (2 h) was 1373 cm^3^·s^−1^. The initial sorption speed of TiZrVAl sample 1# was 1473 cm^3^·s^−1^, and the final sorption speed was 1273 cm^3^·s^−1^. The initial sorption speed of TiZrVAl sample 2# was 2073 cm^3^·s^−1^, and the final sorption speed was 1973 cm^3^·s^−1^.The initial sorption speed of TiZrVAl sample 3# was 1873 cm^3^·s^−1^, and the final sorption speed was 1673 cm^3^·s^−1^. The sorption speeds of TiZrVAl 2# and TiZrVAl 3# were significantly higher than that of TiZrV. TiZrVAl 2# displayed the best H_2_ gettering performance, with the highest sorption speed and the slowest attenuation. The initial sorption speed of TiZrVAl 3# was slightly lower than that of the TiZrVAl 2# sample, and the gettering performance decay was also faster.

**Figure 11:**
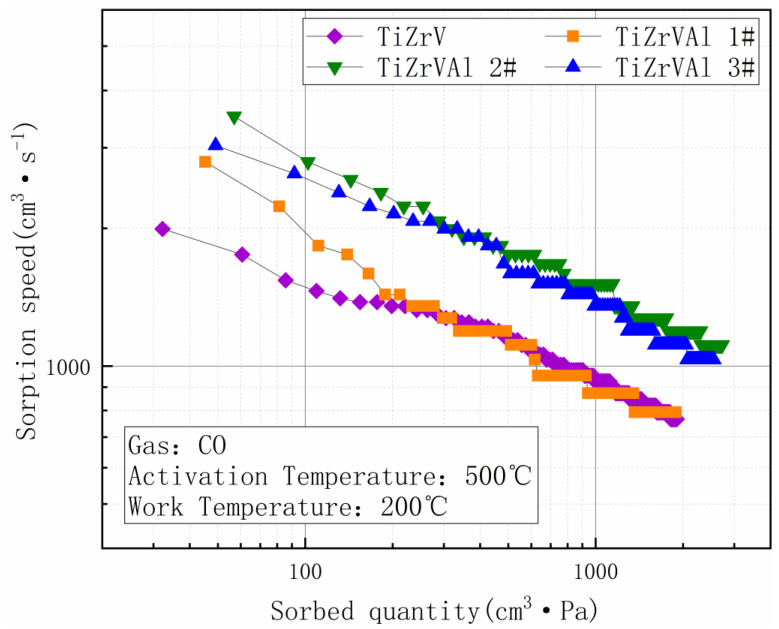
Performance of absorbing CO of TiZrV and TiZrVAl 1–3#.

Figure 11 shows the CO gettering performance of the samples, which were activated at 500 °C and tested at 200 °C. The initial sorption speed of the TiZrV sample at 10 min was 1326.13 cm^3^·s^−1^, and the final sorption speed at the end of the test (2 h) was 766.13 cm^3^·s^−1^. The initial sorption speed of TiZrVAl sample 1# was 1352.8 cm^3^·s^−1^, and the final sorption speed was 792.8 cm^3^·s^−1^. The initial sorption speed of TiZrVAl sample 2# was 1912.8 cm^3^·s^−1^, and the final sorption speed was 1112.8 cm^3^·s^−1^.The initial sorption speed of TiZrVAl sample 3# was 1912.8 cm^3^·s^−1^, and the final sorption speed was 1032.8 cm^3^·s^−1^. Compared to the TiZrV sample, TiZrVAl sample 1# showed a slightly higher gettering performance in the initial stage and gradually decayed to a similar sorption speed. TiZrVAl samples 2# and 3# exhibited a comparable performance; their sorption speeds were close, and the decay situation was similar. The decay trend of the posterior segment was relatively similar in all the samples.

**Figure 12:**
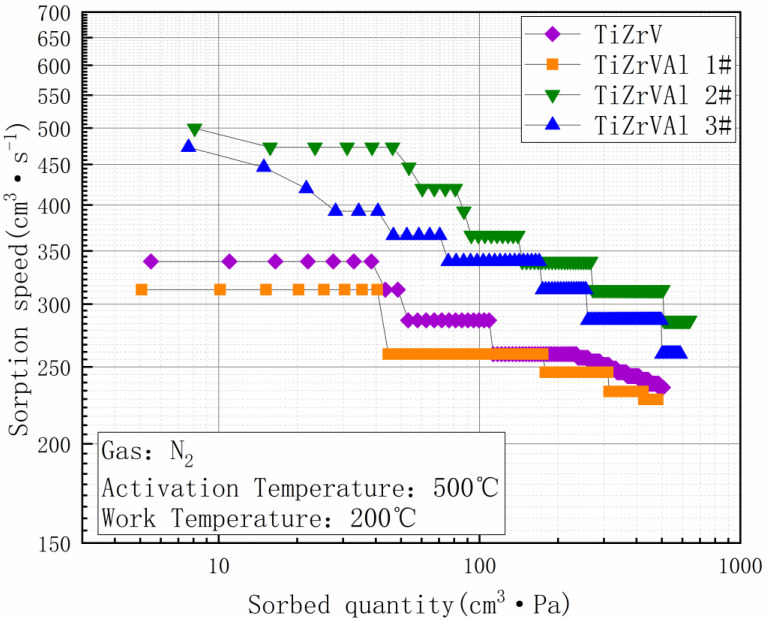
Performance of absorbing N_2_ of TiZrV and TiZrVAl 1–3#.

The gettering performance of N_2_ is shown at Figure 12. The activation conditions and test temperature of the samples were the same as the conditions in the test of H_2_. The performance of the TiZrV getter in absorbing N_2_ was significantly lower than that of absorbing CO. The difference should be related to the bond energy of the gases. The chemical bond of N_2_ is a nitrogen triple bond, resulting in a higher chemical stability than that of the carbon–oxygen bond in CO. The initial sorption speed of the TiZrV sample at 10 min was 286.13 cm^3^·s^−1^, and the final sorption speed at the end of the test (2 h) was 235.47 cm^3^·s^−1^. The initial sorption speed of TiZrVAl sample 1# was 259.47 cm^3^·s^−1^, and the final sorption speed was 227.47 cm^3^·s^−1^. The initial sorption speed of TiZrVAl sample 2# was 419.47 cm^3^·s^−1^, and the final sorption speed was 286.13 cm^3^·s^−1^.The initial sorption speed of TiZrVAl sample 3# was 366.13 cm^3^·s^−1^, and the final sorption speed was 259.47 cm^3^·s^−1^. The initial sorption speed of TiZrVAl sample 1# with a small amount of Al added was slightly lower than TiZrV. However, it significantly reduced and was lower at the end of the test. The initial sorption speeds of TiZrVAl 2# and 3# were significantly higher than that of TiZrV during the whole test process. The sorption speed of TiZrVAl sample 2# was the highest among the samples, and it had a similar attenuation amplitude to sample 3# in the second half of the test.

**Figure 13:**
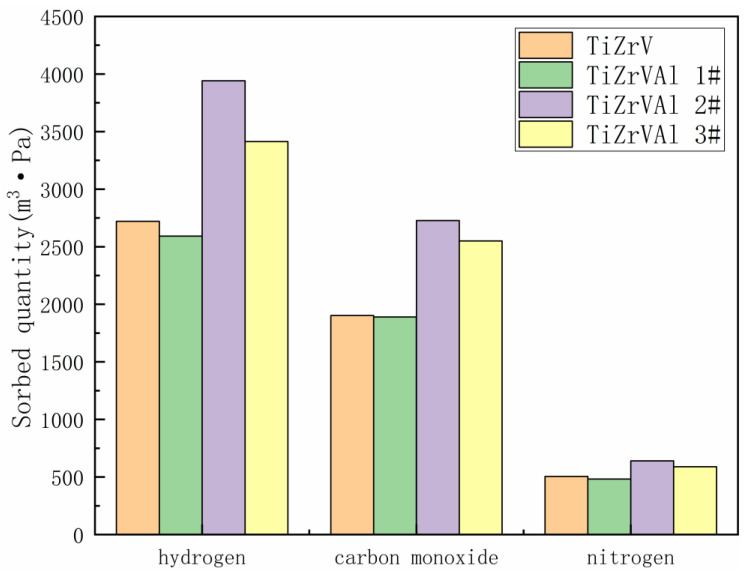
Sorbed quantity of TiZrV and TiZrVAl 1–3#.

Figure 13 shows the sorbed quantity of the TiZrV and TiZrVAl 1–3# samples. It demonstrates the ability of gettering gases, which is related with operating life of getter materials. The sorbed quantity of the samples exhibited a similar pattern to the sorption speed. The performance order was as follows: TiZrVAl 2# > TiZrVAl 3# > TiZrV > TiZrV 1#.

## 4. Discussion

The gettering performance of TiZrVAl samples is significantly better than that of TiZrV, which should benefit from appropriate Al addition. As shown in the results of in situ XPS, the metallic state of the alloy element appeared earlier in TiZrVAl sample 2#. This means that, in the same time and heating conditions, the O, C, and N impurities on the surface of getter materials diffuse into the bulk phase more quickly, indicating a better activation efficiency. In the gettering performance test, TiZrVAl sample 2# showed the best performance for the absorption of the gases. The CO and N_2_ absorption tests were conducted at 200 °C, indicating that the C and O element diffusion in TiZrVAl sample 2# was also significantly easier than that of TiZrV at this temperature.

The gettering performance of ZrVAl sample 2# should benefit from changes in microstructure. The addition of Al elements significantly changed the phase structure of the TiZrV sample. The addition of Al enables the α-Zr phase to transform into Al-Zr and Ti-Zr alloy phases, among which the Al-Zr phase has a larger gap size, which can reduce the diffusion activation energy of elements such as H and O, reducing their diffusion difficulty [26,27]. A better diffusion ability can bring excellent gettering performance, reduce the difficulty of the activation process, lower the activation temperature, and improve activation efficiency.

Compared to TiZrVAl sample 2#, TiZrVAl 3# displayed a respectable CO gettering performance and acceptable H_2_ gettering performance, which should be related to the content of Al and Al-Zr phases. Excessive Al elements exist in the alloy as single elements. During the activation process, trace amounts of aluminum will evaporate and become surface impurities that interfere with gas adsorption. Hydrogen is mainly absorbed and stored in the form of solid solutions and metal hydrides, but the Al element did not have similar characteristics, leading to the degradation of the H_2_ gettering performance [28,29]. As CO was mainly stored in alloys with the form of metal oxides and metal carbides, further improvements in the Al content had little impact on the performance of CO absorption. Moreover, the evaporation characteristics of Al elements is unacceptable for vacuum devices and vacuum apparatus, which limits the application of TiZrVAl getter 3# in vacuum systems.

## 5. Conclusions

We have optimized TiZrV gettering properties with the addition of Al. The results indicate the best performance of samples supplemented with 7 at% Al: the H_2_ gettering performance reaches 2073 cm^3^·s^−1^, increased by 40.7% compared to TiZrV; the CO gettering performance reaches 1912.8 cm^3^·s^−1^, increased by 44.2%; and the N_2_ gettering performance reaches 419.47 cm^3^·s^−1^, increased by 46.6%. The main phases of Al-added samples contained TiZr, Al-Zr, and Al-Ti. The lattice gap of the Al-Zr phase is larger than that of the α-Zr phase, which is conducive to the diffusion of H, O, and other elements. Excessive Al reduced hydrogen storage and deteriorated the gettering performance. Changing the content of phases conducive to diffusion and storage can provide an approach to designing getter materials suitable for different working scenarios.

## Figures and Tables

**Table 1:** The proportion of TiZrV and TiZrVAl 1–3# samples.

	Zr (at%)	Ti (at%)	V (at%)	Al (at%)
TiZrV	50	20	30	-
TiZrVAl 1#	50	20	30	3
TiZrVAl 2#	50	20	30	7
TiZrVAl 3#	50	20	30	11

**Table 2:** Elemental ID and quantification of silicon wafer with coating.

Sample	Name	Start BE	Peak BE	End BE	FWHM eV	Atomic %
TiZrVAl 3#	O1s	537.50	531.85	523.00	2.97	31.18
	C1s	292.50	284.79	277.00	2.63	58.44
	Al2p	79.50	74.14	64.50	2.72	7.25
	Ca2p	356.50	347.28	340.00	2.97	3.13
TiZrVAl 2#	O1s	538.50	531.78	523.00	3.03	27.17
	C1s	292.00	284.71	277.50	1.79	55.47
	Si2p	106.00	99.02	93.50	2.91	17.37

**Table 3:** The ICP-ms test results of impurities in the raw materials.

Sample	Ca (W%)	Zn (W%)	Mg (W%)	Al (W%)
Zr	<0.2	<0.002	<0.002	<0.0021
Ti	<0.001	<0.001	<0.001	/
V	<0.003	<0.001	<0.001	/
Al	<0.001	<0.001	<0.001	/

**Table 4:** EDS selected area scans for TiZrVAl 2#.

	A (at%)	B (at%)
Zr	61.56	38.05
Ti	29.83	18.67
V	6.19	33.99
Al	2.29	9.29

## Data Availability

Data are contained within the article.
